# ﻿Whole-genome based phylogeny and comparative genomics of Sporidiobolales and related taxa of *Basidiomycetes*

**DOI:** 10.3897/imafungus.16.141626

**Published:** 2025-05-13

**Authors:** Yuuki Kobayashi, Naoto Tanaka, Minenosuke Matsutani, Yuuna Kurokawa, Keita Aoki, Moriya Ohkuma, Ri-ichiroh Manabe, Masako Takashima

**Affiliations:** 1 Laboratory of Yeast Systematics, Tokyo NODAI Research Institute (TNRI), Tokyo University of Agriculture, 1-1-1 Sakuragaoka, Setagaya, Tokyo 156-8502, Japan Tokyo University of Agriculture Tokyo Japan; 2 Department of Molecular Microbiology, Faculty of Life Sciences, Tokyo University of Agriculture, 1-1-1 Sakuragaoka, Setagaya, Tokyo 156-8502, Japan Japan Collection of Microorganisms, RIKEN BioResource Research Center Ibaraki Japan; 3 NODAI Genome Research Center, Tokyo University of Agriculture, 1-1-1 Sakuragaoka, Setagaya, Tokyo 156-8502, Japan Laboratory for Comprehensive Genomic Analysis, RIKEN Center for Integrative Medical Sciences Kanagawa Japan; 4 NODAI Culture Collection Center, Tokyo University of Agriculture, 1-1-1 Sakuragaoka, Setagaya, Tokyo 156-8502, Japan Tokyo University of Agriculture Tokyo Japan; 5 Japan Collection of Microorganisms, RIKEN BioResource Research Center, 3-1-1 Koyadai, Tsukuba, Ibaraki 305-0074, Japan Japan Collection of Microorganisms, RIKEN BioResource Research Center Ibaraki Japan; 6 Laboratory for Comprehensive Genomic Analysis, RIKEN Center for Integrative Medical Sciences, 1-7-22 Suehiro-cho, Tsurumi-ku, Yokohama City, Kanagawa, 230-0045, Japan Laboratory for Comprehensive Genomic Analysis, RIKEN Center for Integrative Medical Sciences Kanagawa Japan

**Keywords:** Basidiomycete yeast, comparative genomics, phylogenetic analysis, red yeasts

## Abstract

*Sporidiobolales* is a fungal order of *Basidiomycota* within the subphylum Pucciniomycotina. This order encompasses significant yeasts, such as the oleaginous species *Rhodotorulatoruloides* and the opportunistic pathogen *R.mucilaginosa*. We present the sequencing and comparative analysis of 35 *Sporidiobolales* strains from 27 species, alongside a *Leucosporidium* strain (*Leucosporidiales*), and incorporating publicly available genomic data for related fungi. Based on the phylogenomics data, we found that the topologies obtained were relatively similar and in line with previous reports. A comparison between genomic makeup and previously described phenotypes revealed that the ability to utilize nitrate, raffinose, rhamnose, or sucrose clearly correlated with the existence of key enzymes involved in the corresponding metabolic pathways. However, similar associations could not be established for other carbon sources, such as maltose, galactose, or xylose. We further identified orthologs that are specifically present or absent in each taxon. These results and the genomic sequencing data will help in gaining a better understanding of these non-model yeast species.

## ﻿Introduction

Yeasts are unicellular fungi widely distributed on earth. Since the first discovery of basidiospore formation in yeast (Banno 1967), it has become clear that there are many lineages that form a yeast stage in *Basidiomycota* (Kurtzman et al. eds. 2011). The first yeast species described as *Basidiomycota* is one of the most common red yeasts, *Rhodotorulatoruloides* (Banno 1967). *R.toruloides* is classified as a species of the class *Microbotryomycetes* in the subphylum Pucciniomycotina (Kurtzman et al. 2011). *Microbotryomycetes* consists of orders including the smut fungi *Microbotryales*, the white yeasts *Leucosporidiales*, and the red yeasts *Sporidiobolales* (Wang et al. 2015a, b). *Sporidiobolales* was previously classified as two ballistospore-forming genera: the anamorphic genus *Sporobolomyces* and the corresponding teleomorphic genus *Sporidiobolus*, as well as two non-ballistospore-forming genera: the anamorphic genus *Rhodotorula* and the corresponding teleomorphic genus *Rhodosporidium* (Kurtzman et al. eds. 2011). Reflecting subsequent phylogenetic studies and the change of nomenclature code, which integrated anamorphic and teleomorphic names, *Sporidiobolales* is currently divided into three genera: *Sporobolomyces*, *Rhodotorula*, and *Rhodosporidiobolus* (Wang et al. 2015a, b). At present, more than fifty species of *Sporidiobolales* have been reported (Li et al. 2020) including lipid-producing species such as *R.toruloides* (Ageitos et al. 2011) and opportunistic pathogens such as *R.mucilaginosa* (Jarros et al. 2020). Therefore, it is valuable to study these yeasts from both industrial and clinical perspectives.

Because yeasts have fewer morphological characteristics compared to multicellular organisms such as plants and animals, the current classification of yeasts is mainly constructed by molecular methods. The internal transcribed spacer (ITS) sequences of the ribosomal RNA (rRNA) precursor gene in combination with D1-D2 sequences of the 26S rRNA large subunit (LSU) have been primarily used for taxon identification and phylogenetic studies of yeasts (Kurtzman and Robnett 1997; Scorzetti et al. 2002). For a more comprehensive phylogenetic assessment, the sequences of conserved housekeeping genes, such as those encoding RNA polymerases, translation elongation factors, and cytochrome B, have been used (Baldauf et al. 2000; James et al. 2006; Kurtzman 2014). In recent years, technological advances in genome sequencing have enabled the use of whole-genome information for taxonomic studies. Whole-genome sequencing data are now being used for phylogenetic classification of taxa through so-called phylogenomic approaches in fungi (Riley et al. 2016; Choi et al. 2017; Shen et al. 2020). However, for basidiomycete yeasts, this approach has been limited to a few studies, such as those on *Trichosporonales* (Takashima et al. 2019) and *Malassezia* (Wu et al. 2015).

In addition to phylogenetic assessment, whole-genome sequencing has revealed many biological characteristics of yeasts. In contrast to the model yeast *Saccharomycescerevisiae*, whose genome information is widely used in basic biology (Cherry et al. 1998), most genome research on yeast-forming basidiomycetes is directed towards practical applications. One notable example is the human pathogen *Cryptococcusneoformans*, whose genome has been extensively studied, yielding critical insights for clinical diagnostics and therapeutic developments (Heitman et al. 1999; Idnurm et al. 2005). Genomic studies on the opportunistic pathogen *Trichosporonasahii* compared a clinical strain (Yang et al. 2012a) with an environmental strain (Yang et al. 2012b).

Genomic research on *Sporidiobolales* initially centered on the oil productivity of *R.toruloides*. The NP11 strain was initially sequenced to unravel the lipid biosynthesis pathways (Zhu et al. 2012). Subsequently, the genome of the ATCC 204091 strain, which was previously identified as *R.glutinis*, was sequenced to provide a genomic data resource for another oil-producing strain (Paul et al. 2014). The IFO 0559 and IFO 1236 strains were then sequenced for the establishment of transformation systems to produce high lipid-producing strains (Zhang et al. 2016). The genome of the IFO 0880 strain was sequenced to produce targeted gene deletion lines for investigating metabolic pathways (Coradetti et al. 2018). In addition to the oil-producing species *R.toruloides*, the genomes of *R.graminis* WP1 strain, an endophyte that enhances plant growth isolated from a poplar stem (Firrincieli et al. 2015), and *R.taiwanensis* MD1149 strain, isolated as a radiation-resistant strain (Tkavc et al. 2018), have been sequenced and reported. However, a comprehensive genome analysis of multiple species across the entire order has not yet advanced.

In this study, we sequenced genomes of fungi belonging to *Sporidiobolales* species from the Japan Collection of Microorganisms (JCM), RIKEN BioResource Research Center (BRC), and the National Biological Resource Center, Japan (NBRC) with the objective of compiling their genome information. Together with publicly available genome sequences, we inferred phylogenetic relationships based on whole-genome information by means of different methods. We compared the repertoire of metabolic genes to described phenotypes such as carbohydrate assimilation and searched for orthologous genes that characterize each taxon to find markers for identification.

## ﻿Materials and methods

### ﻿Fungal cultures and sequencing

All strains sequenced in this study were obtained from the Japan Collection of Microorganisms (JCM), the RIKEN BioResource Center (BRC), Tsukuba, Japan, and the National Biological Resource Center (NBRC), National Institute of Technology and Evolution (NITE), Kisarazu, Japan (Table 1). Strains were incubated for one to five days in YM liquid medium (10 g glucose, 3 g yeast extract, 3 g malt extract, and 5 g peptone per litre) at 25 °C, with shaking at 100 rpm.

**Table 1.** Summary of assembly genomes.

Species	Strain	Estimated genome size (bp)	Total assembly size (bp)	No. of contigs	Average length (bp)	Max length (bp)	N50	BUSCO completeness (fungi_odb10, n = 758)				
								Complete	Single	Duplicated	Fragmented	Missing
*R.glutinis*	JCM 8208^T^	21,383,036	21,031,453	544	38,660.8	412,935	124,170	79.9	79.6	0.3	5.4	14.7
*R.graminis*	JCM 3775^T^	20,228,226	20,429,315	362	56,434.6	557,532	160,126	77.8	77.7	0.1	6.1	16.1
*R.babjevae*	JCM 9279^T^	20,980,473	21,042,041	369	57,024.5	595,387	178,254	84.2	83.9	0.3	4.5	11.3
*R.araucariae*	JCM 3770^T^	19,161,872	19,688,143	815	24,157.2	190,232	51,923	83.7	83.6	0.1	4.1	12.2
*R.paludigena*	JCM 10292^T^	20,643,218	20,813,848	177	117,592.4	853,605	293,322	89.7	89.4	0.3	2.6	7.7
*R.sphaerocarpa*	JCM 8202^T^	17,478,281	17,460,028	221	79,004.7	1,019,573	173,083	86.5	86.4	0.1	3.6	9.9
*R.mucilaginosa*	JCM 8115^T^	20,611,328	20,115,778	343	58,646.6	606,815	125,031	87.6	87.6	0.0	2.8	9.6
*R.dairenensis*	JCM 3774^T^	19,296,451	19,455,747	534	36,434.0	256,967	79,538	85.7	85.4	0.3	4.4	9.9
*R.pacifica*	JCM 10908^T^	20,492,110	20,374,152	102	199,746.6	1,534,540	562,053	88.8	88.5	0.3	2.6	8.6
*R.toruloides*	JCM 10020	20,645,899	20,492,593	88	232,870.4	1,672,916	976,034	86.7	85.5	1.2	3.6	9.7
*R.toruloides*	JCM 10021	20,781,719	20,389,818	78	261,407.9	1,973,210	938,736	88.2	87.9	0.3	3.0	8.8
*R.toruloides*	JCM 10049	20,313,569	20,270,281	53	382,458.1	1,516,062	930,903	85.7	85.2	0.5	4.7	9.6
*R.toruloides*	JCM 24501	20,174,084	20,321,374	38	534,773.0	1,645,103	1,204,811	88.3	87.9	0.4	1.7	10.0
*R.toruloides*	NBRC 10512	20,153,584	20,381,470	324	62,905.8	464,688	168,846	86.8	86.4	0.4	3.3	9.9
*R.toruloides*	NBRC 10513	21,662,728	20,583,880	155	132,799.2	1,266,296	475,791	88.5	88.4	0.1	2.8	8.7
*Rhodotorulaazoricus*	JCM 11251^T^	22,839,766	22,682,430	300	75,608.1	603,599	223,031	89.1	89.1	0.0	2.1	8.8
*Rhodotorulamicrosporus*	JCM 6882^T^	25,074,185	25,687,078	608	42,248.5	437,949	101,261	85.7	85.4	0.3	3.7	10.6
*Rhodotorulalusitaniae*	JCM 8547^T^	24,604,526	24,563,961	510	48,164.6	546,650	135,555	87.7	87.7	0.0	2.8	9.5
*Rhodotorularuineniae*	JCM 8097	25,069,789	25,142,625	409	61,473.4	765,765	188,449	87.8	87.5	0.3	2.8	9.4
*Rhodotorulapoonsookiae*	JCM 10207^T^	23,993,186	23,978,565	374	64,113.8	575,888	167,670	86.2	85.9	0.3	4.1	9.7
*Rhodotorulaodoratus*	JCM 11641^T^	26,022,727	25,070,242	911	27,519.5	431,847	98,890	88.8	88.4	0.4	1.7	9.5
*Rhodotorulanylandii*	JCM 10213^T^	24,785,751	24,737,636	726	34,073.9	473,705	128,444	85.9	85.5	0.4	4.2	9.9
*S.johnsonii*	JCM 1840^T^	22,015,142	21,811,286	422	51,685.5	698,303	136,638	79.9	79.9	0.0	5.5	14.6
*S.johnsonii*	JCM 5296	23,744,835	21,533,936	815	26,422.0	190,683	54,066	80.6	80.3	0.3	5.1	14.3
*S.salmonicolor*	JCM 1841^T^	19,818,358	20,466,144	785	26,071.5	259,920	50,696	80.6	80.5	0.1	5.9	13.5
*S.salmonicolor*	JCM 21900	19,507,291	20,162,018	1,018	19,805.5	131,956	37,030	78.6	78.5	0.1	7.0	14.4
*S.pararoseus*	JCM 3765	20,731,455	21,161,000	463	45,704.1	658,648	124,825	91.9	91.6	0.3	1.5	6.6
*S.pararoseus*	JCM 5350^T^	22,872,948	22,408,324	938	23,889.5	333,600	57,498	90.4	90.0	0.4	2.5	7.1
*S.salmoneus*	JCM 6883	20,539,372	20,345,533	264	77,066.4	962,155	285,613	90.8	90.8	0.0	1.5	7.7
*S.carnicolor*	JCM 3766^T^	19,484,300	19,514,829	526	37,100.4	450,810	91,840	88.4	88.4	0.0	3.8	7.8
*S.ruberrimus*	JCM 16303	21,017,201	20,920,594	275	76,074.9	804,554	212,308	89.2	89.1	0.1	1.8	9.0
*S.phaffii*	JCM 11491^T^	19,158,692	19,322,758	463	41,733.8	449,409	105,658	88.1	88.1	0.0	3.7	8.2
*S.koalae*	JCM 15063^T^	18,341,066	18,348,248	157	116,867.8	1,700,008	371,635	90.5	90.5	0.0	1.6	7.9
*S.roseus*	JCM 5353^T^	23,222,652	23,327,241	1,591	14,662.0	165,583	29,640	89.8	89.7	0.1	2.8	7.4
*S.blumeae*	JCM 10212^T^	20,156,654	19,940,609	814	24,497.1	300,686	53,586	84.0	83.9	0.1	4.5	11.5
*L.creatinivora*	JCM 10699	27,665,643	27,969,159	122	229,255.4	1,866,479	836,129	89.0	87.9	1.1	2.6	8.4

T: Type strain.

From *R.toruloides* strains JCM 10020, JCM 10021, JCM 10049, JCM 24501 and *L.creatinivora* (JCM 10699), cells were freeze-dried and crushed with a mortar and pestle, genomic DNA was extracted according to Raeder and Broda (1985) and purified with Genomic-tip 100/G columns (Qiagen, Netherlands); Illumina libraries with insert sizes of approximately 240 bp were constructed with a TruSeq DNA PCR-free (Illumina, USA) preparation kit, and the libraries were paired-end sequenced using the HiSeq 2500 (Illumina, USA). In the case of the other 31 strains (Table 1), genomic DNA was extracted after crushing the cell wall mechanically with a mortar and pestle and, following a protocol optimized for Westase enzyme (Takara, Japan), and genomic libraries with insert sizes of 350 bp were constructed with an Illumina DNA Prep, (M) Tagmentation kit (Illumina, USA), and the libraries were paired-end sequenced on a NextSeq 500 (Illumina, USA). For all libraries, sequences of 151 bp were obtained.

### ﻿Genome assembly, assessment, and annotation

The genome sizes were estimated with GenomeScope1.0 (Vurture et al. 2017) following k-mer (k = 21) counting with Jellyfish 2.3.0 (Marcais and Kingsford 2011). Draft genomes were assembled with Allpath-LG v52155 (Gnerre et al. 2011) for JCM 10020, JCM 10021, JCM 10049, JCM 10699, and JCM 24501, and with SPAdes genome assembler v3.15.3 (Prjibelski et al. 2020) for the other strains. Completeness of assembled genomes was assessed with BUSCO 5.4.2 (Manni et al. 2021) with fungi_odb10 (n = 758) selected as the reference database.

Genes were predicted with MAKER 2.31.8 (Cantarel et al. 2008) using the combination of GeneMark-ES 4.21 (Lomsadze et al. 2005) and Augustus 3.0.3 (Stanke et al. 2006) for JCM 10020, JCM 10021, JCM 10049, JCM 10699, and JCM 24501, and with BRAKER 2.1.6 (Bruna et al. 2021) using the combination of GeneMark-ES 4.69 (Lomsadze et al. 2005) and Augustus 3.4.0 (Stanke et al. 2008). The protein dataset of RefSeq *R.toruloides* NP11 (GCF_000320785.1, Zhu et al. 2012) was used as an Augustus hint.

### ﻿Phylogenetic analyses

For the construction of a concatenated BUSCO single-copy tree, conserved single-copy orthologs were searched from protein sequences with BUSCO 5.4.2 (Manni et al. 2021) with fungi_odb10 (n = 758) selected as the reference database. The sequences of 331 BUSCO orthologs found as complete single copies in all strains were extracted with SeqKit 2.2.0 (Shen et al. 2016). Concatenated sequences were aligned with MAFFT 7.508 (Katoh et al. 2013). The phylogenetic tree was constructed with IQ-tree 2.2.0.3 (Nguyen et al. 2015) with “-m MFP -B 1000 --alrt 1000 --abayes -T 8” options. The gene concordance factors (gCF) and site concordance factors (sCF) were calculated using the WAG amino acid model and executed with the default commands, following the instructions on the IQ-tree website. For the construction of a tree integrating trees for all orthologous genes, OrthoFinder 2.5.4 (Emms and Kelly 2019) was used. For this analysis a species tree was obtained with STAG (Emms ane Kelly 2018) while the integrated gene tree was rooted with STRIDE (Emms and Kelly 2017), both of which were included in the OrthoFinder execution. All applications were run with default options. The distance-based phylogenetic tree with Balanced minimum evolution (BME) support values was constructed with JolyTree 1.1b (Criscuolo 2019; Criscuolo 2020) using default options.

### ﻿Assessment of gene diversity of the genus

The common orthogroup (OG) score was calculated following the description in Takashima et al. (2019), using OrthoFinder 2.5.4 (Emms and Kelly 2019) to identify OGs. The pairwise average amino acid identity (AAI) was calculated using CompareM v. 0.1.2 (https://github.com/dparks1134/CompareM) with default parameters. The shared OG rate was calculated based on the results obtained from OrthoFinder 2.5.4 (Emms and Kelly 2019).

### ﻿Assignment of KEGG ontology and comparative analyses

The amino acid sequences of predicted genes were assigned to KEGG orthologs (KOs) using the GhostKOALA web server (Kanehisa et al. 2016, accessed Aug 3, 2023). The enzymes involved in the conversion of each nutrient were identified from KEGG metabolic pathway maps obtained via the KEGG Mapper website (Kanehisa and Sato 2020, accessed Aug 3, 2023). The physiological traits referred to were based on the findings of Kurtzman et al. (2011), Satoh and Makimura (2008), and Huang et al. (2011).

### ﻿Reference genomes

Assembly genomes and gene catalogs used as reference, i.e. *R.toruloides* NP11 (Zhu et al. 2012), *R.toruloides* IFO 0559 (Zhang et al. 2016), *R.toruloides* IFO 1236 (Zhang et al. 2016), *R.toruloides* IFO 0880 v4.0 (Coragetti et al. 2018), *R.toruloides* ATCC 204091 (Paul et al. 2014), *R.taiwanensis* MD1149 (Tkavc et al. 2018), *R.graminis* WP1 (Firrincieli et al. 2015), *L.creatinivorum* UCDFST 62-1032 (Mondo et al. 2017), *M.intermedium* 1389 BM 12 12 (Branco et al. 2017), *M.lychnidis-dioicae* p1A1 Lamole (Perlin et al. 2015), were downloaded from the JGI MycoCosm web portal (Grigoriev et al. 2014). Details are summarized in Suppl. material 1: table S1.

### ﻿Abbreviations

**AAI**: average amino acid identity

**BRC**: BioResource Research Center

**ITS**: internal transcribed spacer

**JCM**: Japan Collection of Microorganisms

**KEGG**: Kyoto Encyclopedia of Genes and Genomes

**KO**: KEGG ortholog

**LSU**: large subunit

**NBRC**: National Biological Resource Center

**OG**: orthogroup

**rRNA**: ribosomal RNA

## ﻿Results

### ﻿General features of Sporidiobolales genomes

We sequenced 35 yeast strains from 27 species of *Sporidiobolales* and a *Leucosporidium* strain, maintained in RIKEN BRC-JCM and NBRC, including type strains of nine *Rhodotorula*, six *Rhodosporidiobolus*, and eight *Sporobolomyces* species (Table 1). Expected genome sizes were determined by k-mer analysis and estimated for *Sporidiobolales* to be between 17 and 26 Mbp whereas that of *L.creatinivorum* was approximately 28 Mbp (Table 1). The predicted genome sizes of *Rhodosporidiobolus* were slightly bigger than those of *Rhodotorula* and *Sporobolomyces*. The k-mer histograms showed a single peak for each genome, suggesting that these strains have a haploid karyotype (Suppl. material 1: fig. S1), although for a few strains such as *S.pararoseus*JCM 5350 and *S.roseus*JCM 5353 small satellite peaks were observed, which can be caused by sequence errors or aneuploidy.

In line with the predicted genome sizes, the genome assemblies for *Sporidiobolales* were between 17 and 26 Mbp, and that of *L.creatinivorum* was approximately 28 Mbp (Table 1). These genome sequences are also similar to those of the reference genome (Suppl. material 1: table S1). Also, genome-assembly sizes of *Rhodosporidiobolus* were slightly bigger than those of the other *Sporidiobolales*. The comprehensiveness of the genomes assessed with BUSCO was between 78% and 92% with up to 1.2% duplicated genes, suggesting that these genome assemblies represent haploid cells (Table 1).

Homology-based gene prediction estimated that *Sporidiobolales* strains have approximately 6,400 to 9,800 genes, while *L.creatinivorum* has 10,454 genes (Table 2). Consistent with the assembly size, the gene numbers of *Rhodosporidiobolus* were relatively bigger than those of *Rhodotorula* and *Sporobolomyces*. The completeness of the BUSCO assessment was 93% to 97% with up to 1.3% duplicated genes, also suggesting that these gene data are highly comprehensive and indicative for haploid gene sets (Table 2).

**Table 2.** Summary of predicted genes.

Species	Strain	No. of genes	BUSCO completeness (fungi_odb10, n = 758)				
			Complete	Single	Duplicated	Fragmented	Missing
*R.glutinis*	JCM 8208^T^	7,882	95.3	94.9	0.4	1.7	3.0
*R.graminis*	JCM 3775^T^	7,626	96.3	96.2	0.1	1.3	2.4
*R.babjevae*	JCM 9279^T^	7,741	96.3	96.0	0.3	1.1	2.6
*R.araucariae*	JCM 3770^T^	7,482	94.6	94.2	0.4	1.7	3.7
*R.paludigena*	JCM 10292^T^	7,753	97.2	97.1	0.1	1.1	1.7
*R.sphaerocarpa*	JCM 8202^T^	6,408	95.8	95.8	0.0	1.5	2.7
*R.mucilaginosa*	JCM 8115^T^	7,155	96.3	96.3	0.0	1.2	2.5
*R.dairenensis*	JCM 3774^T^	6,885	93.1	93.0	0.1	3.0	3.9
*R.pacifica*	JCM 10908^T^	7,406	97.1	96.8	0.3	0.5	2.4
*R.toruloides*	JCM 10020	8,047	96.8	95.5	1.3	0.8	2.4
*R.toruloides*	JCM 10021	8,007	96.7	96.0	0.7	1.2	2.1
*R.toruloides*	JCM 10049	7,938	96.8	96.3	0.5	0.7	2.5
*R.toruloides*	JCM 24501	8,001	96.8	96.4	0.4	0.7	2.5
*R.toruloides*	NBRC 10512	8,254	97.5	97.0	0.5	0.7	1.8
*R.toruloides*	NBRC 10513	8,308	97.5	97.0	0.5	0.4	2.1
*Rhodotorulaazoricus*	JCM 11251^T^	8,028	96.8	96.4	0.4	1.1	2.1
*Rhodotorulamicrosporus*	JCM 6882^T^	9,769	95.3	94.9	0.4	1.7	3.0
*Rhodotorulalusitaniae*	JCM 8547^T^	9,421	97.4	97.0	0.4	0.7	1.9
*Rhodotorularuineniae*	JCM 8097	9,586	96.2	95.9	0.3	1.5	2.3
*Rhodotorulapoonsookiae*	JCM 10207^T^	9,313	96.4	96.0	0.4	1.2	2.4
*Rhodotorulaodoratus*	JCM 11641^T^	8,476	94.8	94.3	0.5	1.6	3.6
*Rhodotorulanylandii*	JCM 10213^T^	9,337	96.9	96.2	0.7	1.1	2.0
*S.johnsonii*	JCM 1840^T^	7,701	96.8	96.7	0.1	1.3	1.9
*S.johnsonii*	JCM 5296	7,614	94.4	94.3	0.1	1.2	4.4
*S.salmonicolor*	JCM 1841^T^	7,111	96.1	96.0	0.1	1.7	2.2
*S.salmonicolor*	JCM 21900	6,992	94.3	94.3	0.0	2.2	3.5
*S.pararoseus*	JCM 3765	7,917	96.3	96.0	0.3	1.2	2.5
*S.pararoseus*	JCM 5350^T^	8,374	95.9	95.6	0.3	1.2	2.9
*S.salmoneus*	JCM 6883	7,662	97.1	97.0	0.1	0.7	2.2
*S.carnicolor*	JCM 3766^T^	7,215	93.6	93.3	0.3	2.8	3.6
*S.ruberrimus*	JCM 16303	7,444	96.5	96.4	0.1	0.9	2.6
*S.phaffii*	JCM 11491^T^	7,249	95.6	95.5	0.1	1.2	3.2
*S.koalae*	JCM 15063^T^	6,593	96.6	96.2	0.4	0.7	2.7
*S.roseus*	JCM 5353^T^	9,067	95.3	94.6	0.7	2.0	2.7
*S.blumeae*	JCM 10212^T^	7,167	93.9	93.8	0.1	2.5	3.6
*L.creatinivora*	JCM 10699	10,454	97.2	96.0	1.2	0.4	2.4

^T^: Type strain.

### ﻿Phylogenetic analyses from whole genome information

**Figure 1.**
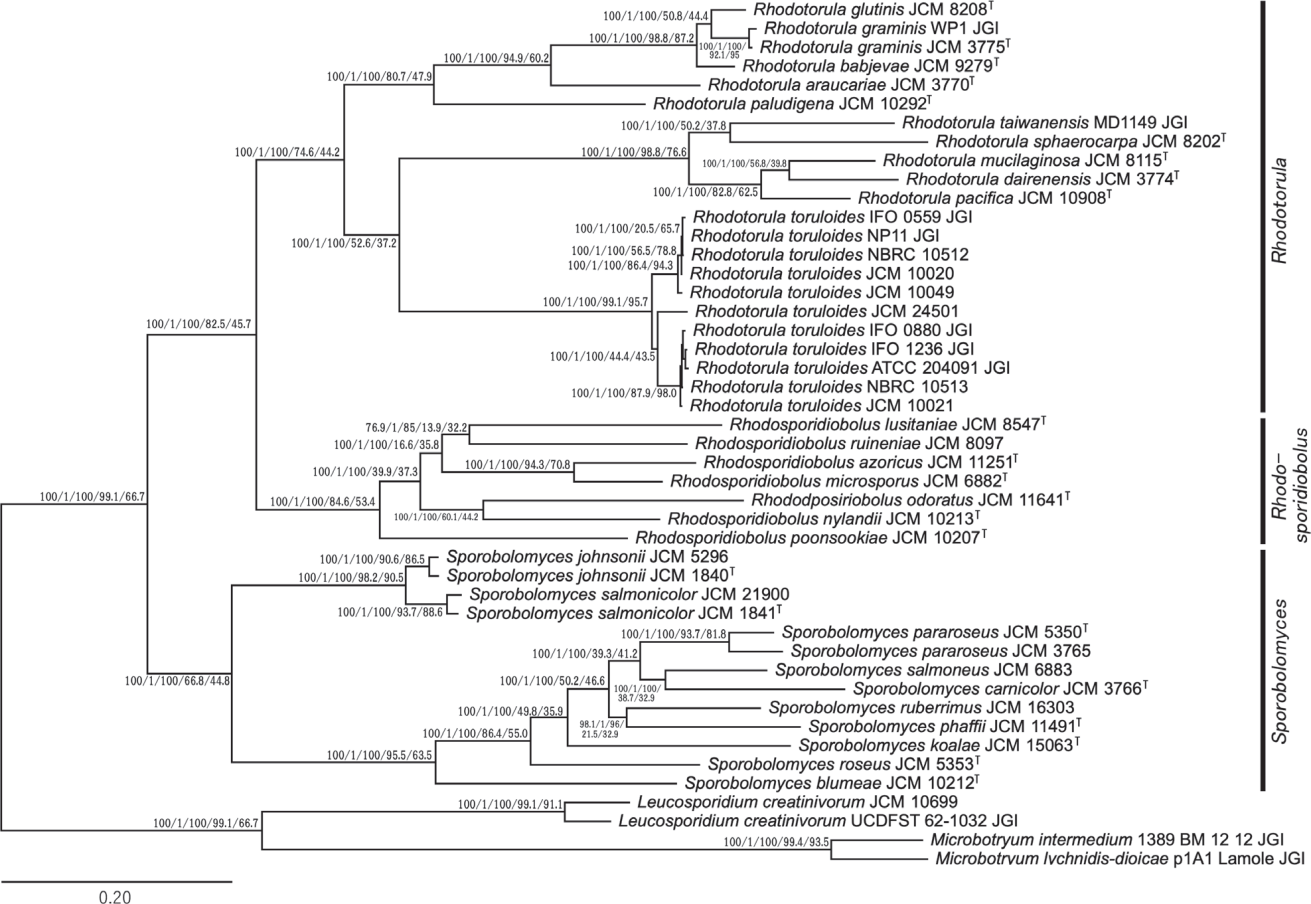
A phylogenetic tree based on concatenated protein sequences of 331 BUSCO genes found as single copy in all strains. The numbers beside the nodes represent the reliability based on SH-aLRT, aBayes, ultrafast bootstrap values, gene concordance factors (gCF), and site concordance factors (sCF) from left to right.

**Figure 2.**
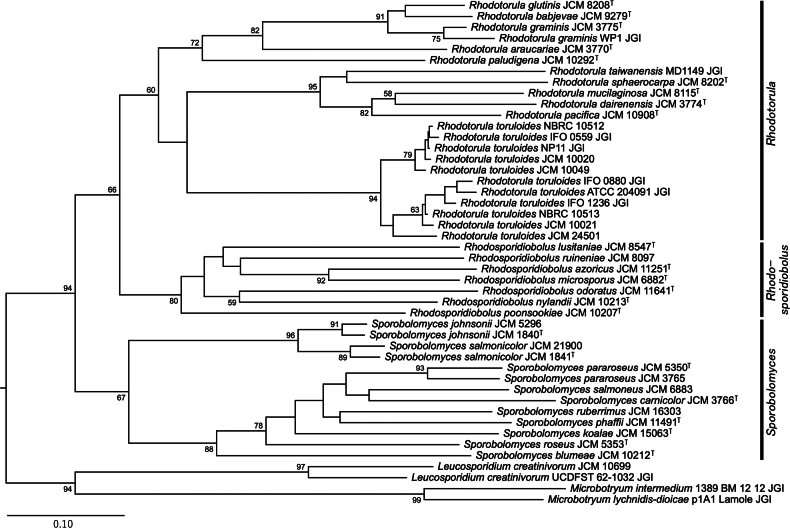
A phylogenetic tree inferred from gene trees of 3,181 orthologs found in all strains. The root is estimated with STRIDE. The numbers beside the nodes represent STAG support values.

**Figure 3.**
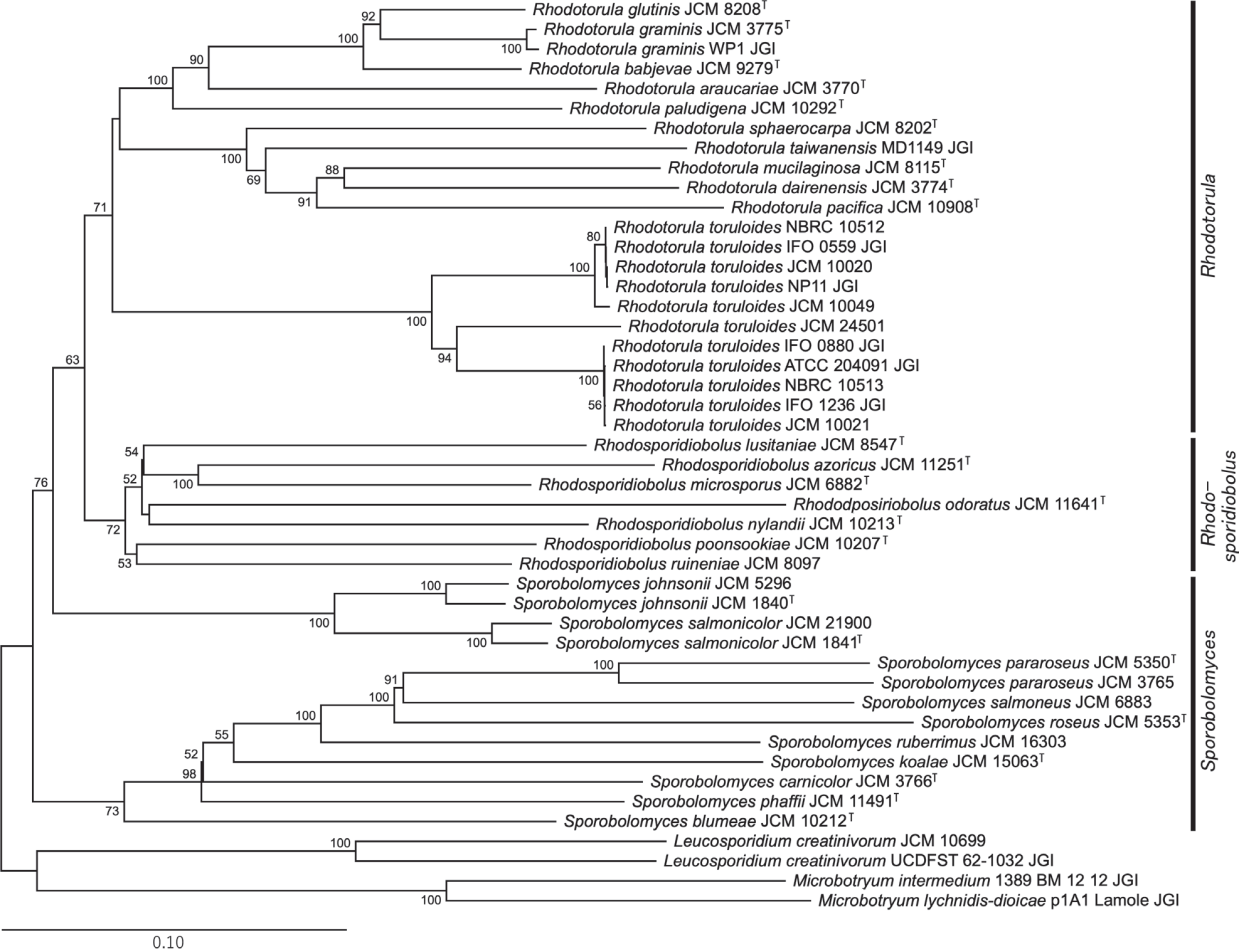
A phylogenetic tree based on the similarity of whole-genome nucleotide sequences. The numbers beside the nodes represent balanced minimum evolution (BME) support values.

We predicted the phylogenetic relationships between these strains from whole-genome data, including *Sporidiobolales* and other *Microbotryomycetes* (*Leucosporidium* and *Microbotryum*) the data for which were retrieved from the JGI Mycocosm (Grigoriev et al. 2014). We constructed phylogenetic trees using three methods: a tree from concatenated protein sequences of BUSCO genes found as a single copy in all strains (Fig. 1), a tree integrating gene trees of all orthologs (Fig. 2), and an alignment-free tree based on the distance of genomic nucleotide sequences (Fig. 3).

The BUSCO assessment of the gene catalog for each species found that 331 BUSCO orthologs (fungi_odb10) were present as single copies in all strains (Suppl. material 1: table S2). A phylogenetic tree was based on the concatenated amino acid sequences of these 331 BUSCO genes and can be considered as a phylogenetic tree of marker genes expanded to the whole-genome level. This tree supported the monophyly of each of the genera *Rhodotorula*, *Rhodosporidiobolus*, and *Sporobolomyces* (Fig. 1). *Rhodotorula* is first divided into two clades: one with *R.glutinis*, *R.graminis*, *R.babjevae*, *R.araucariae*, and *R.paludigena*, and the other with *R.taiwanensis*, *R.sphaerocarpa*, *R.mucilaginosa*, *R.dairenensis*, *R.pacifica*, and *R.toruloides*. The second clade is further divided into the *R.toruloides* clade and others. *Sporobolomyces* can be divided into two clades: one with *S.johnsonii* and *S.salmonicolor*, and the other with *S.pararoseus*, *S.salmoneus*, *S.carnicolor*, *S.ruberrimus*, *S.phaffii*, *S.koalae*, *S.roseus*, and *S.blumeae*. The general topology is similar to the phylogenetic tree based on concatenation of seven marker genes in a previous study (Wang et al. 2015a), suggesting the robustness of the topology when relying on the concatenation of conserved genes regardless of whether the used sequences are those for nucleic acids or proteins. The support values of nodes obtained by bootstrapping, aBayes, or the SH-aLRT method were generally high, although they may have been overestimated, a corollary associated with concatenation-based multi-locus phylogenetic trees (Seo 2008). When reliability was assessed using gene concordance factors (gCF) and site concordance factors (sCF), most major nodes showed gCF values over 60 and sCF values over 40. However, not all values were high; even among branches strongly supported by methods such as bootstrapping, aBayes, and SH-aLRT with very high scores, there were often cases where the gCF and sCF values were low.

Orthologs were retrieved with OrthoFinder, which assigned 361,309 genes to 13,277 orthogroups, of which 3,181 orthogroups were found in all strains. A phylogenetic species tree inferred by orthologous gene trees also supported the monophyly of each of the genera *Rhodotorula*, *Rhodosporidiobolus*, and *Sporobolomyces*, as well as the subclades mentioned above (Fig. 2). This topology is highly similar to the concatenation-based tree (Fig. 1).

The alignment-free phylogenetic tree based on the distance of nucleotide similarity, although keeping the monophyly of *Rhodotorula* and *Rhodosporidiobolus*, resulted in a somewhat different topology (Fig. 3). The monophyly of *Sporobolomyces* was not supported according to this outcome, and the clade including *S.johnsonii* and *S.salmonicolor* was separated from the clade of other *Sporobolomyces*.

### ﻿Gene diversity of genera in *Sporidiobolales*

**Figure 4.**
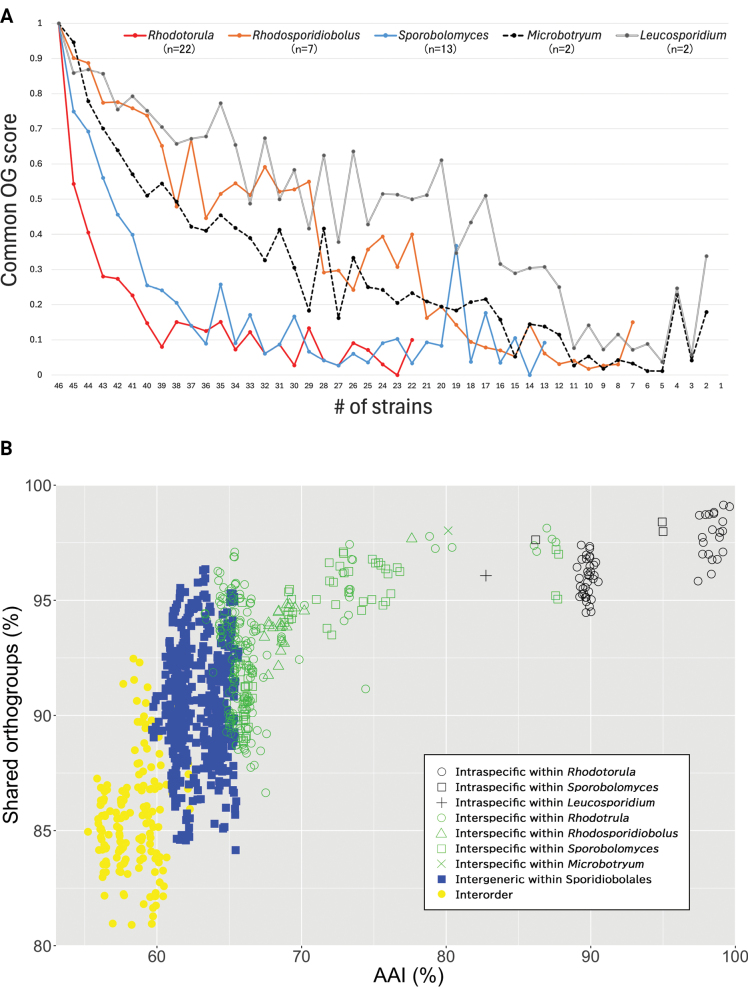
**A** Ratio of conserved orthogroups (OGs) in each genus. The common OG score was calculated as the ratio of the number of OGs maintained in all members of a genus to the total number of OGs, as described by Takashima et al. (2019); **B** plot showing the pairwise average amino acid identity (AAI) similarity and the rate of shared OGs between strains. Open circles, open triangles, open squares, closed squares, and closed circles represent comparisons within *Rhodotorula*, within *Rhodosporidiobolus*, within *Sporobolomyces*, among different genera in *Sporidiobolales*, and among different orders in *Microbotryomycetes*, respectively. The black, green, blue, and yellow markers indicate comparisons within the same species, within the same genus but different species, within the same family but different genera, and among different orders, respectively.

Based on the predicted gene sets, we investigated the gene diversity of genera in *Sporidiobolales*. Initially, we utilized the presence-absence patterns of orthologs (PAPO) method (Takashima et al. 2019). Although the small sample size may have had an impact, the common orthogroup (OG) score of *Rhodosporidiobolus* was the highest among the three genera in *Sporidiobolales* (Fig. 4A). The common OG score of *Rhodotorula* and *Sporobolomyces* was relatively similar and lower than that of *Rhodosporidiobolus*, suggesting that these two genera may exhibit greater ortholog diversity.

Subsequently, we conducted average amino acid identity (AAI) analysis, following the methodology described by Liu et al. 2024. To minimize the influence of gene expansion in specific OGs, we employed the rate of shared OGs instead of shared genes as the y-axis. Similar to the previous study on *Saccharomycetaceae* (Liu et al. 2024), the distribution of AAI values more clearly differentiated taxonomic ranks compared to shared OGs (Fig. 4B, Suppl. material 1: table S3). In *Sporidiobolales*, the AAI values within the same species ranged from approximately 88% to 100%, but the values were calculated based on only 5 species with genome data from multiple strains, including *R.toruloides* (11 strains), *R.graminis* (2 strains), *S.johnsonii* (2 strains), *S.pararoseus* (2 strains), and *S.salmonicolor* (2 strains). The AAI values among different species, but within the same genus, ranged from approximately 65% to 88%, and the AAI values among different genera ranged from approximately 60% to 66%. The lower limit of interspecific AAI within *Rhodotorula* (green open circles in Fig. 4B) was lower than that within *Rhodosporidiobolus* (green open triangles in Fig. 4B) and *Sporobolomyces* (green open squares in Fig. 4B), suggesting that *Rhodotorula* exhibits the most variation in terms of conserved orthologs.

### ﻿Genes contributing to physiological phenotypes

**Figure 5.**
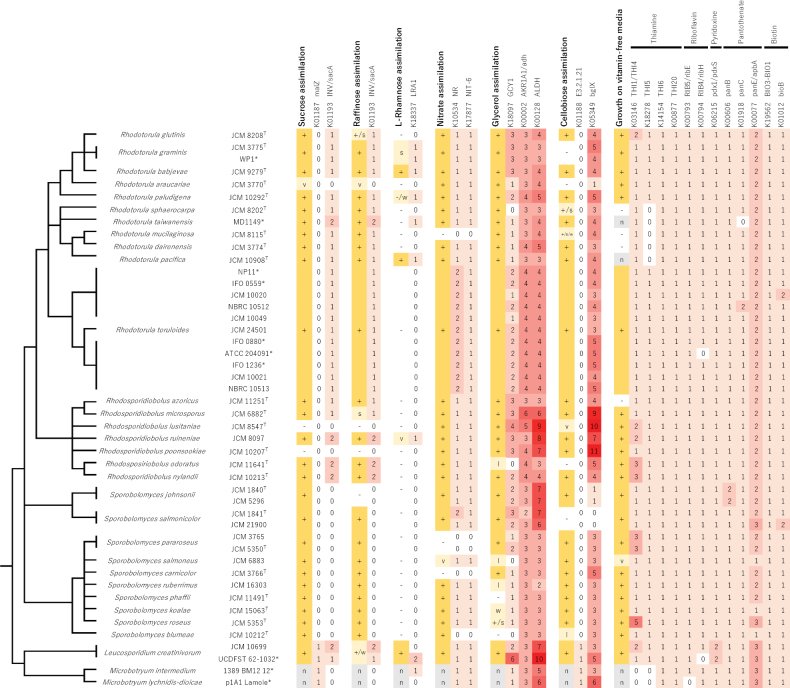
Comparison between assimilation phenotypes and the numbers of metabolic enzymes in the genome for which a correlation is observed. The phenotypes are from Kurtzman et al. eds. (2011), Satoh and Makimura (2008), and Huang et al. (2011), and are indicated as positive (+), negative (-), variable (v), slow (s), latent (l), weak (w), and not available (n), in line with Kurtzman et al. (2011). The phylogenetic tree on the left is the consensus topology of Figs 1–3. Superscripts after strain numbers indicate ‘type strain’ (T), or ‘reference strain’ (*).

Since yeasts have species-specific physiological traits (Kurzman et al. 2010), we examined metabolic genes that contribute to key steps of energy source utilization. We compared gene catalogs derived from sequenced and reference genomes to orthologs annotated in the Kyoto Encyclopedia of Genes and Genomes (KEGG) and counted the number of genes found for each KEGG ortholog (Suppl. material 1: table S4). A clear correlation was observed between the numbers of metabolic genes related to sugar metabolism, nitrogen metabolism, and vitamin biosynthesis with reported physiological traits (Fig. 5). For example, species without the ability to assimilate sucrose and raffinose (*Rhodotorulalusitaniae* and *Rhodotorulapoonsookiae*) lack beta-fructofuranosidase (K01193, INV/sacA), which hydrolyzes sucrose and raffinose into monosaccharides. Species lacking L-rhamnose 1-dehydrogenase (K18337, LRA1) are unable to assimilate L-rhamnose, with the exception of *L.creatinivorum*JCM 10699. *R.mucilaginosa*, *S.pararoseus*, and *S.carnicolor*, which cannot assimilate nitrate, lack both nitrate reductase (K10534, NR) and nitrite reductase (K17877, NIT-6). A clade of *Rhodotorula*, which includes species unable to grow on vitamin-free media (*R.dairenensis*, *R.mucilaginosa*, and *R.sphaerocarpa*), commonly miss the key enzyme for thiamine biosynthesis (K18278, THI5). Species that are unable to fully utilize glycerol as a carbon source (showing negative, weak, or latent growth phenotypes) have no or only one copy of glycerol 2-dehydrogenase (K18097, GCY1), while many strains that grow on media with only glycerol have multiple copies of this ortholog. *S.salmonicolor*, a species lacking beta-glucosidase (K05349, bglX), is unable to convert cellobiose.

**Figure 6.**
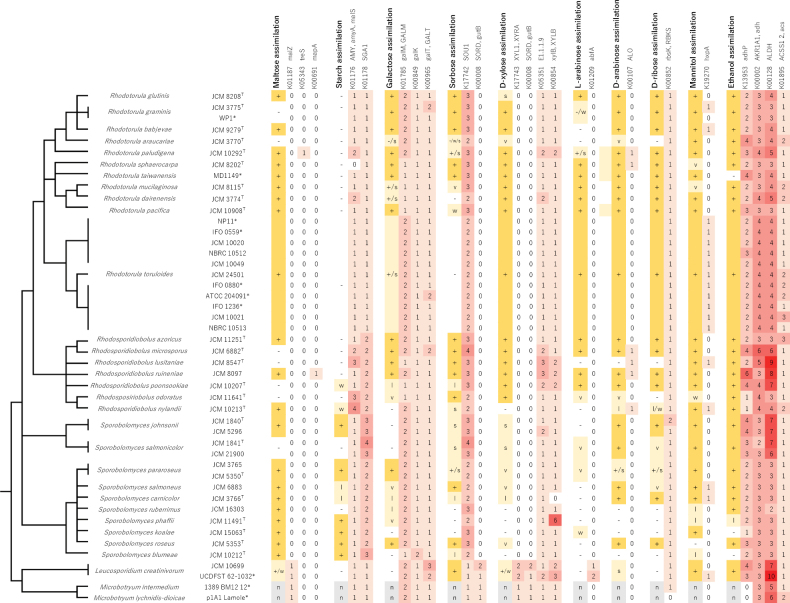
Comparison between assimilation phenotypes and the numbers of metabolic enzymes in the genome for which no clear correlation is observed. See legend of Fig. 5 for details.

For several other carbon sources, however, no correlation was found between the number of genes linked to the associated metabolic pathway and the described phenotypes. This was the case for maltose, starch, galactose, sorbose, xylose, arabinose, ribose, mannitol, and ethanol (Fig. 6). Therefore, not all metabolic traits can be explained by an overlap with KEGG gene sets.

Given the known ability of yeasts in the order *Sporidiobolales* to produce carotenoids such as gamma-carotene, beta-carotene, and torulene (Buzzini et al. 2007), we also examined the copy numbers of genes involved in carotenoid biosynthesis pathways. Carotenoid biosynthesis involves two enzymes: AL1/CAR1 (phytoene desaturase, equivalent to al-1, carB, crtI, K15745), which converts phytoene to 3,4-didehydrolycopene via lycopene, and AL2/CAR2 (bifunctional phytoene synthase/lycopene cyclase, equivalent to al-2, carRA, crtYB, K17841), which converts 3,4-didehydrolycopene to torulene and lycopene to beta-carotene via gamma-carotene (Suppl. material 1: fig. S2 upper panel; Kanehisa and Sato 2020). With the exception of the *S.johnsonii*JCM 5296 strain, which possesses two copies of the AL1/CAR1 gene, all strains of *Sporidiobolales* contain only one copy of each gene involved in carotenoid biosynthesis (Suppl. material 1: fig. S2, lower panel). Thus, differences in carotenoid quantity and composition may depend on the regulation of gene expression rather than the presence of the gene itself.

Several species, such as *R.toruloides* and *R.glutinis*, have been reported to contain high levels of lipids (Chattopadhyay et al. 2021). Therefore, we investigated the lipid synthesis-related genes present in their genomes. By analyzing the number of genes involved in the KEGG pathways for Fatty acid biosynthesis (PATH:ko00061), Fatty acid elongation (PATH:ko00062), and Biosynthesis of unsaturated fatty acids (PATH:ko01040), we found that ACSL/fadD (K01897), which encodes long-chain acyl-CoA synthetase, was slightly more abundant in *R.toruloides* (Suppl. material 1: table S5). However, no other notable differences in gene counts were observed.

### ﻿Taxon-specific KEGG orthologs

To investigate potential marker genes that characterize taxa, we searched for KEGG Orthologs (KOs) that are unique to specific groups. To assess the relationship between gene presence and taxa, we calculated the correlation coefficient between binarized gene presence and taxa. Only KOs identified with an absolute correlation coefficient of 0.7 or higher were considered as relevant. Initially, we analyzed genes that exhibited a varying presence across three orders of *Microbotryomycetes*: *Sporidiobolales*, *Leucosporidiales*, and *Microbotryales* (Suppl. material 1: tables S6–S8). When comparing *Sporidiobolales* with the other two taxa, we identified 43 relevant KOs (Suppl. material 1: table S6), of which 26 were absent in all *Sporidiobolales* strains but present in all the other strains, suggesting they could be potential markers for distinguishing *Sporidiobolales* from other taxa. These include several oligosaccharide hydrolases such as alpha-glucosidase malZ (K01187) and beta-glucosidase E3.2.1.21 (K01188). These functions might be carried out by alternative orthologs like beta-fructofuranosidase INV/sacA (K01193) and beta-glucosidase bglX (K05349) in *Sporidiobolales* (Fig. 5). When comparing two *Leucospodiales* strains with the others, we found 24 KOs that were relevant (Suppl. material 1: table S7). It is noteworthy that both *Leucosporidiales* strains lack the AL1 (K15745) and AL2 (K17841) genes, which are important components of the carotenoid biosynthetic pathway. The absence of these genes in *Leucosporidium* can explain that *Leucosporidium* colonies have a white appearance while *Sporidiobolales* and *Microbotryales* colonies become red when carotenoids accumulate (Wang et al. 2015b). When comparing two *Microbotryales* strains against all the others, we identified 40 KOs (Suppl. material 1: table S8). Despite the limited genome data available for *Microbotryales*, these KOs could potentially serve as markers for distinguishing *Microbotryales* from other taxa.

Next, we searched for genes that are specifically present or absent in one of the three genera of *Sporidiobolales*. In the comparison between *Rhodotorula* and the other two genera, 14 KOs were identified (Suppl. material 1: table S9); ten KOs came out of the comparison between *Rhodosporidiobolus* and the other two genera (Suppl. material 1: table S10), and 25 KOs out of the comparison between *Sporobolomyces* and the other two genera (Suppl. material 1: table S11). Among KOs that differ by presence between all strains of one genus and all strains of the other two genera was ER membrane protein complex subunit 10 (K23570, EMC10), which was absent in *Rhodotorula* but present in *Rhodosporidiobolus* and *Sporobolomyces* (Suppl. material 1: table S9). Protein TBF1 (K22485) was found in *Rhodosporidiobolus* but not in *Rhodotorula* and *Sporobolomyces* (Suppl. material 1: table S10), and uric acid-xanthine permease (K23887, UAPA_C) was absent in *Sporobolomyces* but present in *Rhodotorula* and *Rhodosporidiobolus* (Suppl. material 1: table S11). These KOs can be markers for distinguishing genera among *Sporidiobolales*, while the functional importance of these proteins is unknown.

In addition to comparisons among existing taxa, we checked KOs whose presence differs between the two clades of *Sporobolomyces*. We found 83 relevant KOs, including 4 KOs that are absent in *S.salmonicolor* and *S.johnsonii* but present in all other clades of *Sporobolomyces*, and 21 KOs that are present in *S.salmonicolor* and *S.johnsonii* but not in other *Sporobolomyces* clades (Suppl. material 1: table S12).

We also conducted a search for genes that are indicative of the previous classification, which was based on the presence of ballistospores. Specifically, we examined the presence of certain KOs that exhibited significant differences between the ballistospore-forming group (previously classified as *Sporobolomyces* and *Sporidiobolus*) and the ballistospore non-forming group (previously classified as *Rhodotorula* and *Rhodosporidium*). Despite identifying multiple KOs with absolute correlation coefficients exceeding 0.7, we were unable to find any KO that distinctly distinguished between the two groups (Suppl. material 1: table S13).

Lastly, we conducted a comparative analysis between taxa by focusing on the number of gene components present in specific metabolic pathways or modules. As a result, it was reconfirmed that *Leucosporidium* completely lacks the components of the carotenoid biosynthesis pathway, map00906 (Suppl. material 1: table S14). Additionally, several correlations were observed between each taxonomic group and the number of components they possessed in various metabolic systems (Suppl. material 1: tables S14, S15). However, apart from the carotenoid biosynthesis pathway, no other metabolic systems were found to exhibit differences in multiple components significant enough to suggest substantial alterations in their metabolism. While a detailed examination of the differing genes may eventually lead to functional insights, this remains a task for future research.

## ﻿Discussion

*Sporidiobolales* is a yeast order that consists of three genera, *Sporobolomyces*, *Rhodotorula*, and *Rhodosporidiobolus* (Wang et al. 2015b). In this study, we sequenced the genomes of these yeast strains and examined their phylogenetic relationships based on whole-genome data using three methods. Two of the phylogenetic trees constructed, the concatenated BUSCO gene tree and the integrated orthologous gene tree (Figs 1, 2), showed a roughly identical topology and were comparable to those previously published (Wang et al. 2015a, b), although there were some minor differences. The distance-based phylogenetic tree using whole genome sequences, however, deviated with respect to the prediction that the analyzed *Sporobolomyces* strains did not form a single clade (Fig. 3). This result contradicts prior studies that support the monophyly of *Sporobolomyces*. In particular, a study focusing on mating genes showed that species within *Sporobolomyces* are equally closely related on the phylogenetic tree as those within *Rhodotorula* and *Rhodosporidiobolus* (Coelho et al. 2011). Our examination of OG and AAI supports the notion that the OG diversity of *Sporobolomyces* is not larger than that of *Rhodotorula* (Fig. 4). It is possible that *Sporobolomyces* has undergone diversification in regions beyond orthologous genes, such as non-coding regions, but further investigation is required to ascertain the specifics.

Our phylogenetic trees suggest that certain clades of *R.toruloides* show genetic distances comparable to those observed in other interspecific relationships, such as those involving *R.glutinis*, *R.graminis*, and *R.babjevae* (Figs 1–3). This implies that *R.toruloides* may exhibit higher intraspecific diversity than other species. However, strict species delimitation requires tests based on the biological species concept, including mating compatibility assessments and evidence of gene flow. To clarify the precise species boundaries of *R.toruloides*, we will conduct further analyses focused specifically on this species.

Our phylogenetic trees were based on different methods and resulted in topologies that differed to some extent. In the field of phylogenetics, the concatenation of gene sequences has been widely used in multigene analyses (Rokas et al. 2003). However, it has become clear that concatenation-based methods can have outcomes that are problematic in some cases. For example, concatenated gene trees often become inconsistent with any of the individual gene trees when laterally transferred genes are included (Kubatko et al. 2007; Thiergart et al. 2014). Additionally, bootstrapping scores, even for incorrect topologies, tend to be overestimated for trees based on concatenated sequences (Seo et al. 2008). Hence, several methods have been proposed to infer phylogenetic trees for species by integrating ortholog gene trees, which are also known as coalescent-based methods (Liu et al. 2015; Szöllősi et al. 2015). Besides, alignment-free methods based on the similarity of whole-genome sequences have also been developed (Sims et al. 2009; Criscuolo 2020). To deduce the phylogenetic relationships of yeast, the DNA-DNA reassociation method has also been employed (Sampaio et al. 2001; Gadanho and Sampaio 2002). The process of inferring phylogenetic relationships from the similarity of nucleotide sequences of entire genomes may be regarded as a digitized iteration of such a method. The coalescence-based and genome similarity-based methods are relatively recent, with multiple algorithms having been developed for each concept. Coalescence-based methods are considered to be more robust than concatenation-based approaches (Liu et al. 2015), but the determination of the most suitable method remains inconclusive. The quest for the optimal method extends beyond fungal classification and is also being investigated in relation to diverse taxonomic groups, including plants (Simmons and Gatesy 2015). In our results, the tree topologies from both concatenation-based and coalescence-based methods were similar and aligned more closely with AAI-based evaluations than with those from the distance-based method. Regarding the distance-based method, whole-genome sequences contain many non-coding regions with low conservation. The study that developed this method applied it to phylogenetic analyses at the intrageneric level (Criscuolo 2019); therefore, comparisons at the order level, as in this case, may be less accurate. With regard to the reliability assessment of the concatenation-based method, many branches that did not achieve consensus with other phylogenetic trees exhibited exceedingly high values in the bootstrapping, aBayes, and SH-aLRT evaluations. This phenomenon likely reflects the overestimation previously reported (Seo 2008). However, when assessed using gCF and sCF, many of the non-consensus branches displayed low values. Furthermore, the reliability values for the coalescent tree were often comparable to gCF or situated between gCF and sCF. This suggests that gCF and sCF may be more appropriate than bootstrapping or Bayesian posterior probabilities for evaluating the reliability of multi-gene concatenation-based trees. In any case, the establishment of the optimal method is anticipated, alongside examinations of taxonomic groups, including non-fungal taxa, whose evolutionary process can be corroborated through fossil records.

Our comparison between phenotypes and enzymes involved in the corresponding metabolic pathways (Fig. 5) clearly linked the ability to convert rhamnose to the existence of the RhaI gene. A previous study on *Ascomycota* had shown a similar correlation (Riley et al. 2016). Therefore, the assimilation of rhamnose may be predictable from genomic information regardless of the fungal phylum, *Ascomycota* or *Basidiomycota*, an organism belongs to. The ability to use nitrate as a nutrient was evidently related to the presence of enzymes required for nitrate metabolism, suggesting that this trait may also be predictable from genome data. Similarly, the ability to utilize sucrose and raffinose as carbon sources correlated with the presence of invertase genes in *Rhodotorula* and *Rhodosporidiobolus*. However, invertase genes were not identified in *Sporobolomyces*. The sequence of invertase might be highly divergent in this genus or assimilation of these sugars depends on other enzymes. The ability to convert other carbon sources, including maltose, starch, galactose, sorbose, and xylose, did not show a consistent relationship with the presence of genes known to participate in associated metabolic pathways (Fig. 6). In *Ascomycota* no apparent correlation was found between xylose assimilation and the presence of enzymes expected to be involved in this process (Riley et al. 2016). Further study is needed on the genetic requirements for assimilating these sugars.

In investigating whether orthologs specific to a taxon could be identified, we found several genes whose presence strongly correlates with the taxonomic group they belong to (Suppl. material 1: tables S6–S12). However, no genes were found that could be linked to a phenotypic trait of taxa in *Sporidiobolales* or its subtaxa, although the outgroup *Leucosporidium* is marked by an absence of carotenoid synthase, which is associated with its lack of pigmentation (Suppl. material 1: table S7). Currently, there are no known phenotypes that clearly distinguish the three genera of *Sporidiobolales*. Finding phenotypic differences based on genetic information for these genera remains a challenge for future research.

## ﻿Conclusion

In this study, we sequenced 35 genomes of *Sporidiobolales* and a *Leucosporidium* genome. Our phylogenetic study confirmed the monophyly of the genera *Rhodotorula* and *Rhodosporidiobolus* with all methods tested, while the monophyly of *Sporobolomyces* was supported by two of the three methods. Also, our OG and AAI analyses revealed that strains within each genus are closely related to each other at the orthologous gene level. Comparative studies showed that metabolic traits linked to the usage of several compounds, such as rhamnose and nitrate, can be predicted from gene content, while this is not possible in the case of many other compounds. We identified KEGG orthologs whose presence significantly correlates with each taxon, making them potential taxon markers. These results, along with genome information, may help facilitate a better understanding of *Sporidiobolales* yeasts.

## ﻿Additional information

### Adherence to national and international regulations

All the fungal strains used in this study have been legally obtained, respecting the Convention on Biological Diversity (Rio Convention).

### Author ORCIDs

Yuuki Kobayashi https://orcid.org/0000-0003-1061-3639

Minenosuke Matsutani https://orcid.org/0000-0002-6262-3188

Keita Aoki https://orcid.org/0000-0003-2079-4031

Masako Takashima https://orcid.org/0000-0002-7686-8661

### Data availability

All sequence reads and assemblies were submitted to DDBJ/NCBI under BioProject accession numbers PRJDB3686 (JCM 10020), PRJDB3687 (JCM 10021), PRJDB3688 (JCM 10049), PRJDB3691 (JCM 24501), PRJDB3719 (JCM 10699), and PRJDB17412 (all other strains). The detailed accession numbers for sequence reads and assemblies were described in Suppl. material 1: table S16. Data for phylogenetic analyses were uploaded to FigShare (https://doi.org/10.6084/m9.figshare.25592013).

## Supplementary materials

Supplementary material 1 Images for supplemenatl explanation and tables of full BUSCO tables, KEGG-OGs, taxon-specific genes and accession numbers Data type zip https://binary.pensoft.net/file/1329128This dataset is made available under the Open Database License (http://opendatacommons.org/licenses/odbl/1.0/). The Open Database License (ODbL) is a license agreement intended to allow users to freely share, modify, and use this Dataset while maintaining this same freedom for others, provided that the original source and author(s) are credited.Yuuki Kobayashi, Naoto Tanaka, Minenosuke Matsutani, Yuuna Kurokawa, Keita Aoki, Moriya Ohkuma, Ri-ichiroh Manabe, Masako Takashima
