# Supplementary figures and images for: ﻿Whole-genome based phylogeny and comparative genomics of Sporidiobolales and related taxa of Basidiomycetes
Source: IMA Fungus. 2025 May 13;16:e141626. doi: 10.3897/imafungus.16.141626 (PMC12093110; doi:10.3897/imafungus.16.141626)

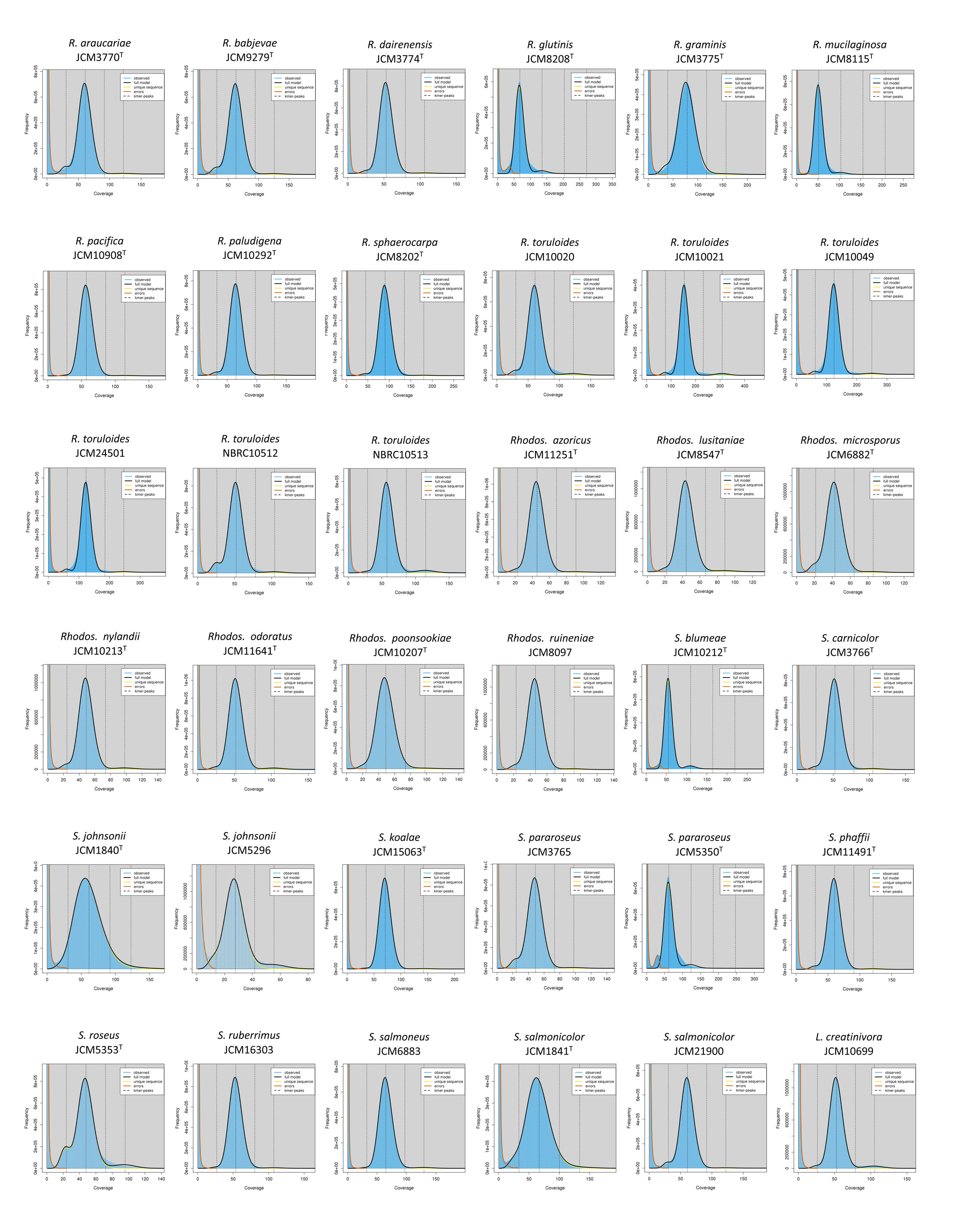

Supplement: Supplementary material 1 — Images for supplemenatl explanation and tables of full BUSCO tables, KEGG-OGs, taxon-specific genes and accession numbers [file imafungus-16-e141626-s001.zip › Supplements/FigS1_GenomeScopeAll.jpg]

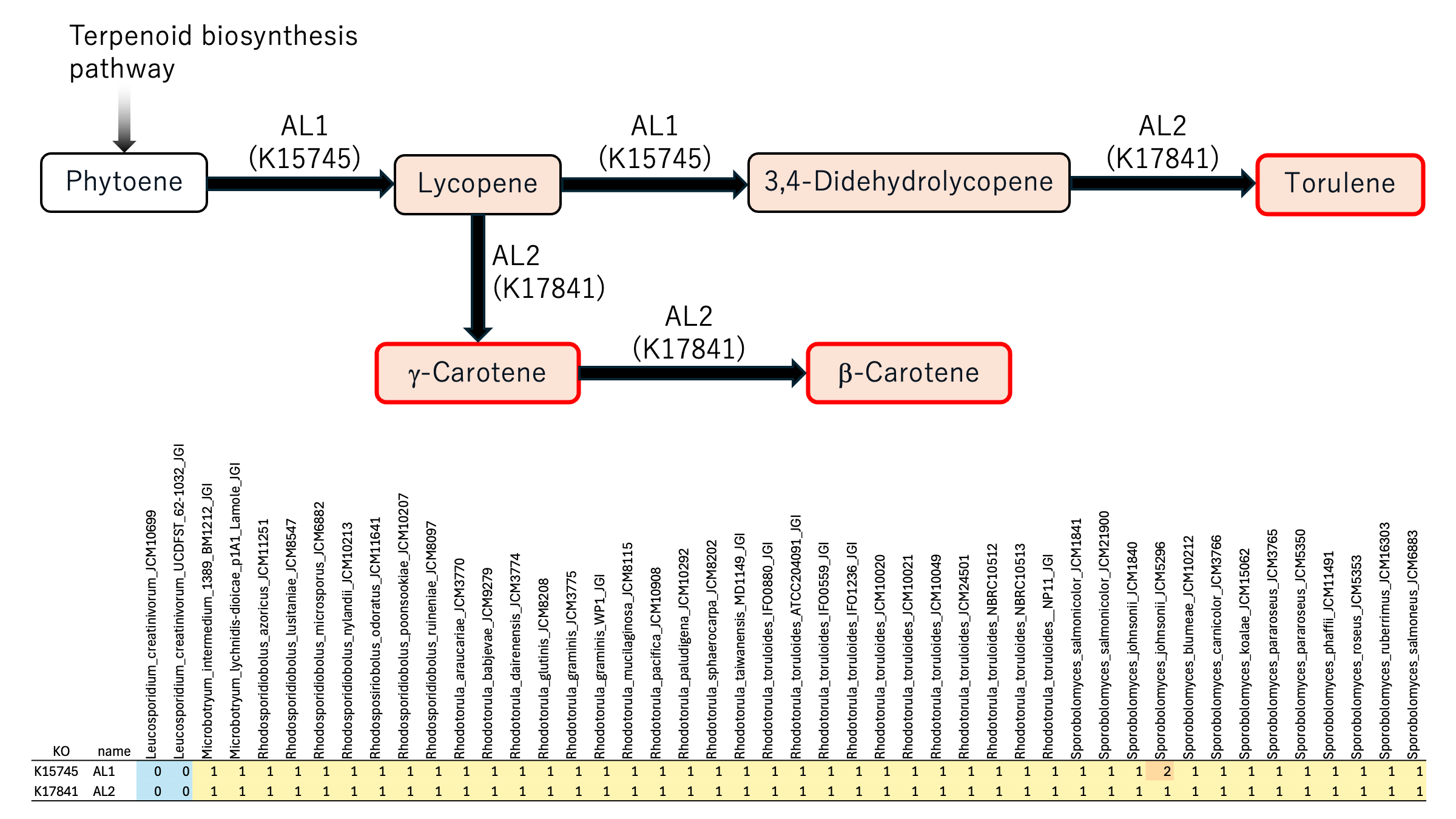

Supplement: Supplementary material 1 — Images for supplemenatl explanation and tables of full BUSCO tables, KEGG-OGs, taxon-specific genes and accession numbers [file imafungus-16-e141626-s001.zip › Supplements/FigS2_CarotenoidKO.png]
